# Beauty‐related perceptual bias: Who captures the mind of the beholder?

**DOI:** 10.1002/brb3.945

**Published:** 2018-03-26

**Authors:** Yan Zhang, Yu Xiang, Ying Guo, Lili Zhang

**Affiliations:** ^1^ School of Educational Science Huazhong University of Science and Technology Wuhan China; ^2^ School of Teacher Education and Psychology Sichuan Normal University Chengdu China

**Keywords:** attractive faces, beautiful flowers, eye tracking, perceptual bias, specificity

## Abstract

**Introduction:**

To explore the beauty‐related perceptual bias and answers the question: Who can capture the mind of the beholder? Many studies have explored the specificity of human faces through ERP or other ways, and the materials they used are general human faces and other objects. Therefore, we want to further explore the difference between attractive faces and beautiful objects such as flowers.

**Methods:**

We recorded the eye movement of 22 male observers and 23 female observers using a standard two‐alternative forced choice.

**Results:**

(1) The attractive faces were looked at longer and more often in comparison with the beautiful flowers; (2) fixation counts of female participants are more than male participants; and (3) the participants watched the beautiful flowers first, followed by the attractive faces, but there was no significant difference on the first fixation duration between the beautiful flowers and the attractive faces.

**Conclusions:**

The data in this study may suggest that people prefer attractive faces to beautiful flowers.

## 1 INTRODUCTION

The human face has been a hot issue in recent years because of the extraordinarily well‐developed technology which could be used to recognize, process, and extract information from others' faces. Several lines of evidence suggest that the neural and perceptual processes involved in face perception in humans are different from those involved in other objects.

One line of evidence is based on studies of face perception in infants. Using a visual tracking task and measuring the time spent by infants looking at different stimuli, several studies have shown that newborn babies (in some cases, a few minutes postpartum) prefer face‐like visual configurations over other, equally complex stimuli (Schwarzer, 2014; Watson, Robbins, & Best, 2014). Another line of evidence suggested that participants were more sensitive to configure changes in face processing than in the visual processing of other objects (Rhodes, 1988; Tanaka & Sengco, 1997). By and large, compared with other objects, face tends to be processed holistically (Farah, Wilson, Drain, & Tanaka, 1998; Tanaka & Farah, 1993). Although occasionally the strategy of holistic processing had been reported on other stimuli, such as on monkey faces (Dufour & Petit, 2010), which have been enhanced in other well‐defined visual categories (Gauthier & Tarr, 1997), only for faces do such strategies develop without special training.

The comparison between the special processing of faces and processing of other objects has been a major debate in visual cognitive neurosciences over the past decades. One aspect of this specificity is the configure processing of faces. The unique relationships among facial features from individual identity, which is not seen in objects that undergo configured processing (Bartlett & Searcy, 1993; Rhodes, Brake, & Atkinson, 1993). Providing excellent temporal resolution of neural events, scalp electrophysiological studies of face processing have focused mainly on negative event‐related potential (ERP) components occurring between 140 and 200 ms after the onset of a stimulus at the occipitotemporal electrodes. This N170 (Bentin, Allison, Puce, Perez, & McCarthy, 1996) component is reliably larger in faces than in any object category tested (Carmel & Bentin, 2002; Itier & Taylor, 2004) and has become a signal for early face processing. Although the exact neural generators of this component are still debated (Watanabe, Kakigi, & Puce, 2003), it is thought to reflect structural encoding in occipitotemporal areas. (Eimer, 2000; Rossion et al., 1999). Many studies have also explored the special process of human faces in recent years (Balas & Saville, 2015; Nemeth, Zimmer, Schweinberger, Vakli, & Kovacs, 2014; Schendan & Ganis, 2013). Balas and Saville demonstrated that the number of faces that one has seen over a lifetime can affect face recognition in adulthood. For women, their beautiful faces are highly correlated with their economic activities (Elder, 1969; Holmes & Hatch, 1938), and the attractive individuals have more chances to have a date than the unattractive (Riggio & Woll, 1984). Beautiful faces arouse more intense positive emotions in observers (Zhang et al., 2011). There are studies indicating that attractive people are thought to be more positive in their personality (Lorenzo, Biesanz, & Human, 2010; Vermeir & Van de Sompel, 2014). Accordingly, attractive people may benefit from the enhanced positivity (Langlois, Kalakanis, Rubenstein, Larson, & Smoot, 2000). In addition, Nemeth, Zimmer, Schweinberger, Vakli, and Kovacs suggested that congenital prosopagnosia is due to the deficit of the early, structural encoding steps of face perception in filtering between face and nonface stimuli. Schendan and Ganis (2013) demonstrated that N170 face specificity is real and cannot be explained by the instantaneous variance or by low‐level psychophysics factors.

In general, people love beautiful things, including beautiful females, handsome males, and attractive natural things. People prefer attractive faces and it is a benefit to be physically attractive, and researchers found the existence of the effect “beauty is good.” Attractive people may benefit from the enhanced positivity (Griffin & Langlois, 2006; Langlois et al., 2000). A study indicated that physically attractive will influence income and financial strain (Judge, Hurst, & Simon, 2009). Besides, the more beautiful one is, the happier he or she will be (Hamermesh & Abrevaya, 2011). In a recent study, the subjects showed different degrees of preference for different types or elements in the landscape (Langlois et al., 2000). They generally preferred natural landscapes, most especially landscape elements such as water and trees, followed by seminatural landscapes and artificial landscapes (Luo, Zhang, Wang, Gan, & Qiu, 2013).

However, only a few studies compared the processing of visual perception between beautiful faces and attractive natural things. In addition, previous experiments mainly adopted event‐related potentials and functional magnetic resonance imaging (fMRI). There are researchers found that compared with fruit and instruments, perception of faces is special (Lavie et al., 2003). Researchers who used event‐related potentials found that the N170 (Bentin et al., 1996) component is reliably larger in faces than in any object category tested (Carmel & Bentin, 2002; Itier & Taylor, 2004) and it has become a sign of early faces processing. The novelty of our study is that we compare the attractive images simultaneously, but other studies compare the general images simultaneously. Thus, we adopted a two‐alternative forced choice paradigm, in which participants were presented simultaneously with two pictures and they had to decide which one he or she thinks is more beautiful. During the process of the EyeLink 1000 eye tracker will record data in their eye movement. Such a paradigm allows testing judgments on stimulus pairs that only differ subtly (Sprengelmeyer et al., 2009). We were interested in the perception of differences between subtle attractive faces and beautiful flowers. Therefore, we adopted stimuli pairs that systematically and independently varied in types and kept them in the same level of attractiveness. The results of previous studies were found in the specificity of human faces. Previous studies mainly compare faces and other objects. This study compares the attractive faces and beautiful flowers, which is aimed to study the specificity processing of attractive faces rather general human faces.

## 2 METHODS

### 2.1 Participants

The participants comprised of 45 volunteers (22 males and 23 females) between 19 and 22 years old. They were all native students in university. Each participant signed an informed consent after the procedure was fully explained. Participants were paid small tokens for their participation. All participants were right‐handed and had normal vision, with no self‐reported history of neurological or psychiatric disorder.

### 2.2 Stimuli

We selected 40 beautiful flowers and 40 attractive faces from a face picture pool (Zhang et al., 2011). First, we collected 845 unfamiliar Chinese female faces from an open picture material resources in the website of Google. There were 490 face stimuli left since low‐resolution images were removed, which was edited to a uniform format. Then, three categories of facial attractiveness (attractive/unattractive/average) were obtained by two specialists in psychology. For the 346 stimuli images selected by the two specialists, a further 9‐step rating on the dimension of attractiveness (a beauty that appeals to the senses of stimuli images), joviality (participants feel jolly and full of good humor when looking at the stimuli images), arousal (a state of heightened physiological activity of participants when looking at the stimuli images), distinctiveness (the degree of distinguishing trait of stimuli images), and 3‐step rating on emotion valence (1—positive, 2—neutral, and 3—negative) were conducted by 80 Chinese college students (mean age 21.98 years) (see Table 1).

**Table 1:** Participants rated different female faces on five dimensions: attractiveness, joviality, arousal, dominance, and emotion valence. The result means that the main effect of attractive category was significantly different (*p *< .001), and the post hoc analyses found that there were significant differences between face categories (all *p *< .01)

Rating dimension (*N *= 80)	Attractive faces, M (*SD*)	Unattractive faces, M (*SD*)	Average faces, M (*SD*)	*F*	*p*
Attractiveness	7.92 (0.82)	2.71 (0.99)	5.35 (1.33)	761.141	.000
Joviality	6.36 (0.68)	2.78 (0.60)	4.36 (0.48)	879.517	.000
Arousal	6.44 (0.71)	6.32 (0.83)	6.26 (0.92)	1.343	.263
Dominance	4.70 (0.46)	4.81 (0.66)	4.77(0.38)	0.996	.370
Emotion valence	2	2	2		

Finally, 84 high attractive face images (rating range: 7–9), 84 low attractive face images (rating range: 1–3), and 168 medium face images (rating range: 4–6) were chosen. The main effect of attractive category was significantly different (*p *<* *.001), and the post hoc analyses found that there were significant differences between face categories (all *p *<* *.01) (see Table 1).

As for the flower images, we also collected 125 flower images from an open picture material resources in the website of Google. Then 68 highly attractive flowers were obtained by two specialists in psychology. For the 68 high stimuli images selected by the two specialists, a further 9‐step rating on the dimension of attractiveness (a beauty that appeals to the senses of stimuli images), joviality (participants feel jolly and full of good humor when looking at the stimuli images), arousal (a state of heightened physiological activity of participants when looking at the stimuli images), distinctiveness (the degree of distinguishing trait of stimuli images), and 3‐step rating on emotion valence (1—positive, 2—neutral, and 3—negative) were conducted by 80 Chinese college students (mean age 21.98 years) .

Finally, 40 highly attractive face images and 40 high attractive flowers images (rating range: 5–9) were chosen. The *t* test effect of all categories was not significantly different (*p *>* *.05) (see Table 2).

**Table 2:** The comparison between flower and female face images. The *t* test effect of all categories was not significantly different (*p *> .05), which means that there is no much difference in the face images and flower images in the attractive level

Rating dimension (*N *= 80)	Faces, M (*SD*)	Flowers, M (*SD*)	*t*	*p*
Attractiveness	6.51 (0.67)	6.58 (0.74)	−0.463	.645
Joviality	6.20 (0.54)	6.10 (0.51)	0.794	.429
Arousal	6.27 (0.60)	6.27 (0.71)	−0.008	.993
Dominance	4.60 (0.51)	4.56 (0.41)	0.442	.660
Emotion valence	2	2		

### 2.3 Procedure

**Figure 1:**
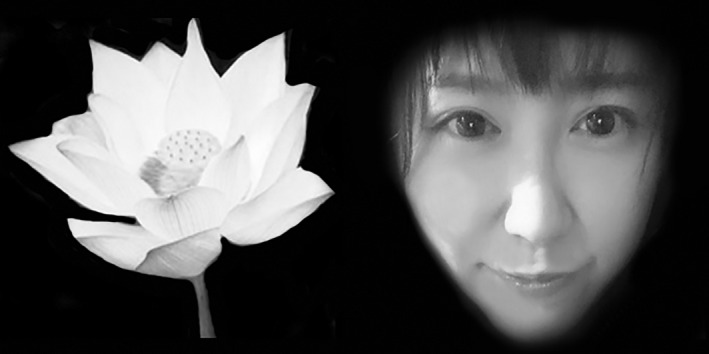
Example of experiment material. It is the images of a beautiful female and a flower

The experiment took place in a dim room. Stimuli were presented on a 17‐inch screen. Eye movements were recorded at a sampling rate of 1000 Hz with an EyeLink 1000 eye tracker. During the experiment, the participants had their helmets and sat approximately 65 cm away from the screen, meaning that each digit occupied 0.95°–1.35° of visual angle. Binocular eye gaze position was recorded during the judgment procedure. Each trial started with a fixation cross. After 1,000 ms, the fixation cross was replaced by a pair of stimuli (see Figure 1). The next pair of stimulus was presented until the participants chose the more attractive one by pressing either the “1” or “2” key on the keyboard (“1” if the left was more attractive and “2” if the right was more attractive) with their respective index fingers. All the participants are right‐handedness, and they all react to the stimulus by the right hands. The location of the two photographs is balanced, which means the time that the one stimulus was presented on the left is equal to the time it was presented on the right.

### 2.4 Statistical analyses

Data were analyzed using SPSS 17.0 for Windows (SPSS Inc, Chicago, IL, USA). The repeated‐measure anova was performed to compare the gender (male/female) as between factors, and areas of interest (attractive faces/beautiful flowers) as a within factor. For all analyses, *p*‐values were corrected for deviation from sphericity according to the Greenhouse–Geisser method. The results section reports the main effects and interactions, which was based on the main hypothesis of the study. Bonferroni post hoc test was conducted when the main effect was significant, and simple effect test was performed when the interaction effect was significant.

The total fixation duration means the time that the participant spends on the object. The longer his eyes stay at the object may indicate that the object is more complicated or that the subject is more interested in the object. The fixation counts means the number of counts his eyes stayed at the object since the participants' eyes will move between the objects. The first fixation location indicates which object the participants look at first. The first fixation duration means how long the participant looks at the first object.

## 3 RESULTS

The total fixation duration was analyzed by repeated‐measure anova with areas of interest (attractive faces/beautiful flowers) and gender (male and female) as factors. There was a significant effect for the areas of interest, *F* (1, 43) = 4.090*, p = *.049, η^2^ *= *0.087*, and* the total fixation time at attractive faces was significantly longer than the beautiful flowers. However, there was no significant effect on gender and no significant effect on interest × gender interaction (see Table 3).

**Table 3:** The eye movement index on beautiful pictures by different participant

Picture Type	Gender	APD	TFT	FFDT	TTFF	FC
Face	M	550.87 ± 175.83	692.96 ± 227.14	281.82 ± 74.82	257.72 ± 81.23	2.46 ± 0.69
	F	474.39 ± 236.90	758.53 ± 293.73	260.50 ± 41.28	341.72 ± 151.65	2.94 ± 1.09
Flower	M	551.37 ± 172.71	567.33 ± 195.23	268.11 ± 64.56	419.72 ± 153.79	1.99 ± 0.62
	F	475.28 ± 230.38	750.37 ± 426.07	270.92 ± 63.53	444.71 ± 189.91	2.67 ± 1.23

There are five eye movement indexes: APD, average pupil diameter; TFT, total fixation time; FFDT, first fixation duration time; TTFF, time to first fixation; FC, fixation count.

The fixation count was analyzed by repeated‐measure anova with areas of interest (attractive faces/beautiful flowers) and gender (male and female) as factors. There was a significant main effect on areas of interest, *F* (1, 43) = 13.224*, p = *.001, η^2^ *= *0.235, and the fixation count at attractive faces was significantly more than the beautiful flowers. And there was a significant effect of gender, *F* (1, 43) = 4.812*, p = *.034, η^2^ *= *0.101, and fixations counts of female participants were more than male participants. However, there was no significant effect on interest × gender interaction (see Table 3).

The first fixation location was analyzed by repeated‐measure anova with areas of interest (attractive faces/beautiful flowers) and gender (male and female) as factors. There was a significant main effect on areas of interest, *F* (1, 43) = 39.057*, p = *.000, η^2^ *= *0.476, and participants watched the beautiful flowers first, followed by the attractive faces. However, there was no significant effect on gender and no significant effect on interest × gender interaction (see Table 3).

The first fixation duration was analyzed by repeated‐measure anova with areas of interest (attractive faces/beautiful flowers) and gender (male and female) as factors. There was no significant main effect for gender, or the areas of interest, and no significant effect on interest × gender interaction (see Table 3).

The average pupil diameter was analyzed by repeated‐measure anova with areas of interest (attractive faces/beautiful flowers) and gender (male and female) as factors. There was no significant main effect for gender, or the areas of interest, and no significant effect on interest × gender interaction (see Table 3).

## 4 DISCUSSION

We found that the total fixation time for attractive faces was significantly longer than that for beautiful flowers and that the fixation count for attractive faces was significantly more than that for beautiful flowers. In the study of Zhang et al. (2011), attractive faces elicited larger early components on P170 and N220 and greater negative amplitude (300–500 ms) compared with unattractive faces at anterior locations, greater negative amplitude (200–300 ms) at temporal and occipital sites, and more late positive components (LPC) at central‐parietal locations. These studies showed that people have a preference for attractive faces. The results are also consistent with the following studies. Earlier fMRI studies in humans have identified an area of the FG implicated in face perception that responds more to faces than to objects (Kanwisher, McDermott, & Chun, 1997; Puce, Allison, Gore, & McCarthy, 1995). This area has been reported to respond similarly to upright and less sensitive to inverted faces (Yovel & Kanwisher, 2004), as participants looked at contrast‐reversed faces (George et al., 1999). The results of the present study are also consistent with those of an early study on face specificity, which adopted the method of ERP (Eimer, 2000), as well as other studies (Ganis, Smith, & Schendan, 2012; Schendan & Ganis, 2013). Ganis, Smith, and Schendan found that N170 face specificity remains even when the interstimulus variance is eliminated. In addition, Schendan and Ganis showed that the N170 peak amplitude and face specificity are quantified for individual stimuli and participants and that the right hemisphere N170 is especially sensitive to stimulus variability, with the interstimulus variance contributing 0% to 37% to N170 face specificity.

Maybe the novelty of the stimuli cannot well explain why people look at faces longer than flowers. In the study of eye movement, the longer fixation means that participants pay more attention to the object for he or she is more interested in the object. Compared with negative‐judgment face, fixation counts are more and fixation time is longer on positive‐judgment face (Kong, 2011). The similar result indicated that longer fixation duration time represents that people are more interested in the object and they are more sensitive to the object, also their process on the object would be further. The result of this study is in accordance with an early study (Kendrick, Atkins, Hinton, Heavens, & Keverne, 1996), which indicated that faces are indeed special for this species, as claimed by human and nonhuman primates. Other studies also showed that familiarity can influence face processing (Barton, Radcliffe, Cherkasova, Edelman, & Intriligator, 2006; Caharel et al., 2007; Kendrick et al., 1996). A recent experiment found that reduced exposure to faces in early life reduces face recognition abilities and neural face specificity at the N170 component (Balas & Saville, 2015).

A potential explanation for people fixating longer and more frequently on the attractive faces than beautiful flowers is the limited resource of attention (Lavie et al., 2003). The result by Lavie, Ro, and Russell showed that face processing may be mandatory and that load theory may be generalized to the processing of meaningful and complex nonface distractors. Using fMRI, we found an area in the FG in 12 of the 15 subjects tested that was significantly more active on faces than assorted common objects (Kanwisher et al., 1997).

In the study, the participants watched the beautiful flowers first, followed by the attractive faces. But there was no significant difference on the first fixation duration between the beautiful flowers and the attractive faces. This finding shows that beautiful flowers may capture the mind of the beholder in the early stage. This result is consistent with our daily experience. In our daily lives, we are often attracted to novel things or to things that we do not always see. Maybe the flowers captured the attention of the observers immediately they look at the pictures. For the flowers are more novel for people than human faces. However, people took more attention on the attractive faces, for they are more familiar with faces, and the attractive faces are of more social value. Therefore, the data in the current study may suggest that people prefer attractive faces to beautiful flowers.

Besides, in this work, fixation was more for the female participants than for the male participants in the selection procedure. Studies have explored perception differences in facial attractiveness among various gender groups. To some extent, these results are inconsistent with those of recent studies (Cloutier, Heatherton, Whalen, & Kelley, 2008; Zhang et al., 2011). The results by Cloutier, Heatherton, Whalen, and Kelley revealed gender differences in the recruitment of OFC, which distinguished attractive and unattractive faces only for male participants. In the study of Zhang et al., the attractive faces elicited larger early components of P170 and N220 and greater negative amplitude (300–500 ms) in comparison with unattractive faces. We only found the gender difference on the fixation counts, no matter what it is, female participants watched more than male participants. It may be related to the gender characteristic that females are more careful than males.

However, previous studies mainly compare faces and other objects, and the present study compares the attractive faces and beautiful flowers, which is aimed to explore the specificity processing of attractive faces rather general human faces. The major contribution of the present study is the finding that facial attractiveness is probably specific. However, some limitations must be considered. First, we did not adopt the attractive faces of male. Second, we did not combine additional methods, such as ERP or fMRI. Finally, we did not explore many types of beautiful things, such as beautiful animals, beautiful buildings, and others.

Collectively, the results of this study showed that people may have a perceptual bias toward attractive faces than toward beautiful flowers.

## AUTHOR CONTRIBUTIONS

Yan Zhang and Ying Guo conceived and designed the experiments, wrote the original draft, analyzed the data, and involved in recruitment and payment of participants. Yan Zhang performed the experiments. Yan Zhang and Lili Zhang wrote the original draft. Yu Xiang, Ying Guo, and Yan Zhang revised the manuscript.

## CONFLICT OF INTEREST

None declared.
