# Beauty‐related perceptual bias: Who captures the mind of the beholder?

**DOI:** 10.1002/brb3.1232

**Published:** 2019-02-18

**Authors:** Yan Zhang, Yu Xiang, Ying Guo, Lili Zhang

In Zhang, Xiang, Guo, and Zhang (2018), the Funding Information field was published with incorrect funder. It should have been “Fundamental Research Funds for the Central Universities, Grant/Award Number: HUST (2017WKYXQN005)”.
